# Variations in the Presence of Carotid Intraplaque Hemorrhage Across Age Categories: What Age Groups Are Most Likely to Benefit From Plaque Imaging?

**DOI:** 10.3389/fneur.2020.603055

**Published:** 2020-12-14

**Authors:** Anthony S. Larson, John C. Benson, Waleed Brinjikji, Luis Savastano, Giuseppe Lanzino, John Huston, Deena Nasr

**Affiliations:** ^1^Department of Radiology, Mayo Clinic, Rochester, MN, United States; ^2^Department of Neurosurgery, Mayo Clinic, Rochester, MN, United States; ^3^Department of Neurology, Mayo Clinic, Rochester, MN, United States

**Keywords:** carotid artery, intraplaque hemorrhage, age, plaque imaging, stroke

## Abstract

Although carotid artery intraplaque hemorrhage (IPH) is a known risk-factor for cerebral ischemic events in patients of advanced age, its prevalence in younger cohorts is less certain. The purpose of this study was to assess the prevalence of carotid artery IPH across the age spectrum. A retrospective review was completed of all adult patients from our institution who underwent neck MRA with high-resolution carotid plaque imaging between 2017 and 2020. The mean ages of patients with and without IPH were calculated. The prevalence of IPH was compared between patients that were categorized into age groups. Patients with and without a cerebral ischemic event (e.g., stroke, retinal ischemia) were included. Unilateral anterior circulation ischemic events in patients without atrial fibrillation were presumed to be likely related to ipsilateral carotid artery disease. Multiple regression analysis was performed to determine independent associations with IPH. 634 patients were included (1,268 carotid arteries). Increasing age (OR: 1.04; 95% CI: 1.02–1.06; *P* = 0.001) was independently associated with IPH. 211 patients had unilateral anterior circulation ischemic events. The mean age of patients with carotid IPH was 71.4 years (SD = 9.9), compared to 62.8 years (SD = 15.8) of those without (*P* ≤ 0.0001). The prevalence of IPH increased with age in all patients (*P* = 0.0002). Among patients with ipsilateral anterior circulation ischemic events, each age category above 50 years had a significantly higher prevalence of IPH when compared to patients 18–50 years (*P* ≤ 0.05 for all comparisons). The prevalence of carotid IPH increases with age and is rare in patients under 50 years. The approximate threshold age for IPH development is likely around 50 years.

## Introduction

Carotid artery atherosclerosis is a well-known risk factor for ischemic stroke (1). With advances in carotid plaque imaging, various plaque characteristics such as intraplaque hemorrhage (IPH), lipid rich necrotic core and inflammation have become increasingly recognized as features of plaque vulnerability (2). IPH, in particular, is a strong predictor of future cerebral ischemic events independent of carotid stenosis or ischemic symptoms (3–5). It is therefore important to be able to recognize which patients may be at risk for IPH so that appropriate intervention may be pursued.

To date, the majority of studies regarding IPH involve cohorts of patients of advanced age. Almost certainly, this is because the risk of both stroke and carotid artery atherosclerosis substantially increases with age (6–8). Nevertheless, it remains uncertain if a threshold age exists at which the prevalence of IPH becomes more prominent. Such information may aid in determining which age groups may be at higher risk for IPH, and therefore would benefit from dedicated carotid plaque imaging. The objectives of the current study were to (1) Identify the prevalence of IPH across the age spectrum in a large cohort of patients, and (2) Determine at which age the prevalence of IPH becomes more prominent.

## Methods

Following Institutional Review Board approval, the medical and imaging records of all adult patients (18 years of age or older) from our institution who underwent neck MRA imaging from 2017 to 2020 were retrospectively reviewed. Patients were excluded if (1) MRAs did not include high-resolution vessel wall imaging with Magnetization Prepared-Rapid Gradient Echo (MPRAGE) sequences (to evaluate for IPH) or (2) images were of suboptimal quality (such as motion artifact). Relevant variables extracted from each record included patient age at MRA imaging, vascular comorbidities and whether or not the patient had a documented history of a cerebral ischemic event defined as ischemic stroke, transient ischemic attack (TIA), retinal artery occlusion (RAO), or amaurosis fugax. All included patients were evaluated by a neurologist, ophthalmologist, or neurosurgeon at our institution.

### Grouping Strategies

Patients were stratified into the following subgroups: (1) asymptomatic patients (no documented history of a cerebral ischemic event as defined above), and (2) symptomatic patients (history of one or more unilateral anterior circulation cerebral ischemic events likely due to carotid artery disease). The symptomatic group excluded patients with posterior circulation strokes, bilateral cerebral ischemic events, or atrial fibrillation. Patients within each subgroup were further divided into the following age categories: 18–50 years, 50–59, 60–69, 70–79, >80.

The majority of patients in the asymptomatic group (88.0%) underwent neck MRA imaging for symptoms thought potentially related to carotid artery pathology (including dissection), but were ultimately not found to have ischemic stroke, TIA, RAO, or amaurosis fugax. A minority of patients (12.0%) underwent neck MRA for evaluation of an underlying connective tissue disease (i.e., Ehlers-Danlos syndrome, Loeys-Dietz syndrome, fibromuscular dysplasia, etc.) or for a history of head/neck neoplasia and/or radiation therapy.

### MR Imaging Protocol

Neck MR imaging was performed as previously described (9). The carotid vessel wall imaging was performed on a 3T MRI scanner (GE 750, GE Healthcare, Milwaukee, WI) with a 16-channel head/neck/spine (HNS) coil and included three sequences: (1) 2D time of flight (TOF); (2) 3D fast spoiled gradient echo acquired in the coronal plane; (3) gadolinium bolus carotid magnetic MRI acquired in the coronal plane. A previously described 3D MPRAGE sequence was used (10) with the following parameters: TR/TE = 13.2 ms/3.2 ms, flip angle = 15°, in plan spatial resolution = 0.63 mm × 0.63 mm, reconstructed resolution = 0.31 mm × 0.31 mm, slice thickness = 1 mm, number of excitation = 2, TI = 304 ms, TR with respect to the non-selective inversion = 568 ms, acquisition time = 3 min 50 s.

### Multiple Logistic Regression Analysis

In order to determine if age was independently-associated with the presence of IPH, multiple logistic regression analysis was performed. Independent variables included age, sex, degree of carotid stenosis and cardiovascular comorbidities (coronary artery disease, hypertension, hyperlipidemia, obstructive sleep apnea, current or former tobacco smoker, diabetes mellitus). The presence or absence of carotid IPH as noted on MPRAGE imaging was the dependent variable. In patients with IPH, the degree of carotid stenosis in the ipsilateral artery was included, whereas in patients without IPH, the artery with the highest degree of stenosis was included in the multiple logistic regression analysis.

### Analysis of Carotid Stenosis and IPH

The following imaging variables were collected from each record: Degree of bilateral carotid stenosis based on North American Symptomatic Carotid Endarterectomy Trial (NASCET) criteria (11), and the presence or absence of IPH (with laterality). IPH was defined as being hyperintense on MPRAGE, with intralesional signal >150% than that of the ipsilateral sternocleidomastoid muscle as assessed by a staff neuroradiologist at our institution. This definition is in accordance with prior literature (4, 5). The degree of stenosis and presence of IPH was determined for each individual carotid artery in patients within the asymptomatic subgroup and in all patients combined. For patients with symptomatic carotid plaques, only the carotid artery ipsilateral to the ischemic event was considered. The prevalence of each degree of carotid stenosis was compared between age categories in each subgroup. The prevalence of IPH was compared between age categories.

### Statistical Analysis

The study primary aim was three-fold: (1) to determine if age is independently associated with the presence of carotid IPH, (2) to determine if there was a difference in age between patients with and without IPH, and (3) to determine the difference in prevalence of carotid artery IPH between age categories within the aforementioned subgroups. Percentages were calculated for binary and categorical variables. Means and standard deviations were calculated for continuous variables. Student's two-tailed *t*-test was used to determine significance between mean ages. Fisher's Exact Probability Test was used to determine significance between the prevalence of IPH compared directly between two age categories. A Chi Square test was used to determine significance between categorical variables including the prevalence of carotid artery severity across age categories as well as the prevalence of IPH across all age categories. Odds ratios (OR) and 95% confidence intervals (CI) were calculated for each variable in the multiple regression analysis. A finding was considered statistically significant for any *P*-value < 0.05. We did not perform any corrections for multiple comparisons. All calculations were performed in Microsoft Excel and Stata statistical software, version 14.1 (StataCorp, College Station, TX, USA).

## Results

### Patients and Carotid Artery Groups

**Figure 1:**
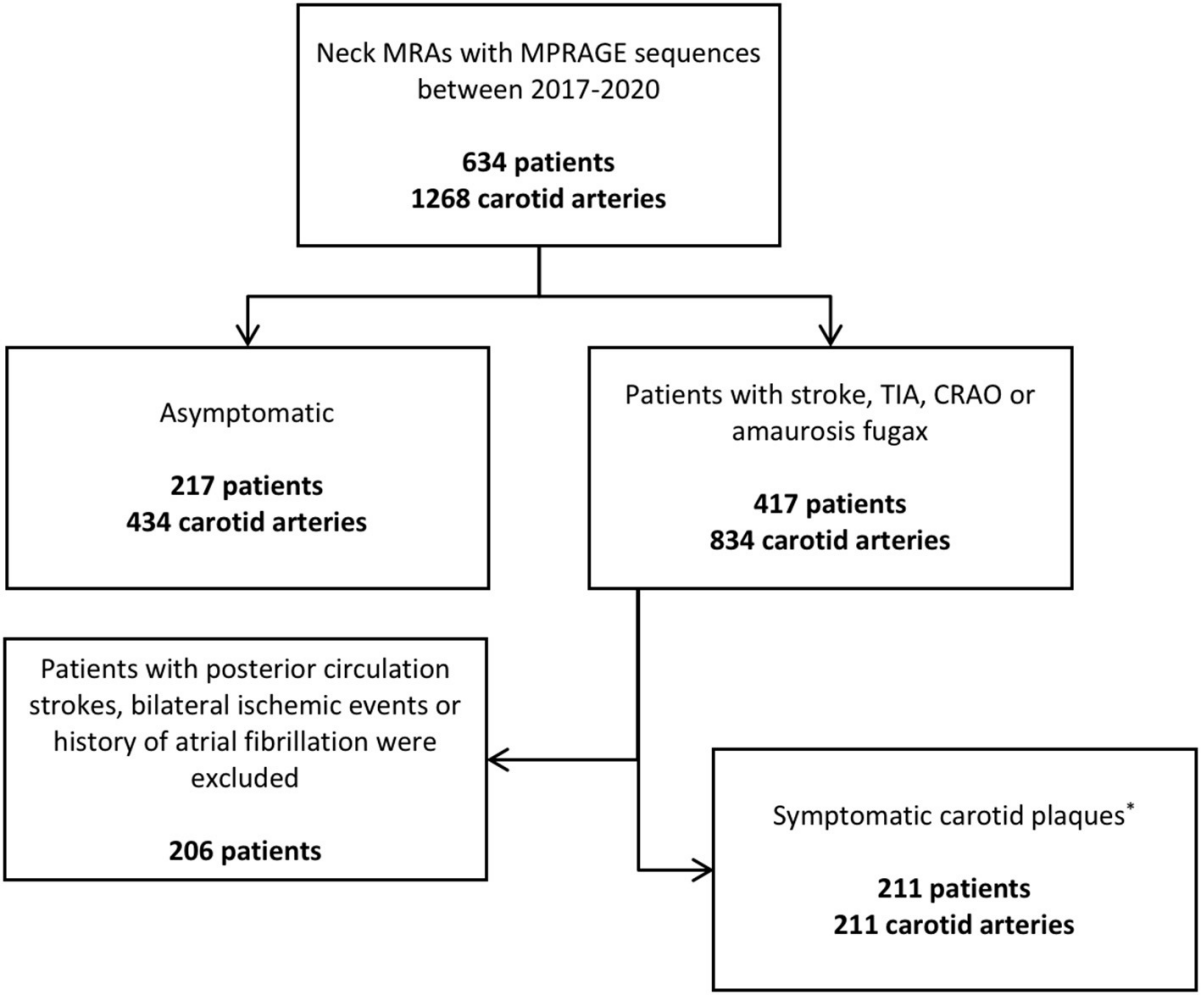
Patient stratification process. *Refers to patients with unilateral anterior circulation ischemic strokes. CRAO, central retinal artery occlusion; MPRAGE, Magnetization Prepared-Rapid Gradient Echo; TIA, transient ischemic attack.

Our patient selection process is outlined in Figure 1. There were 634 patients included, for a total of 1,268 carotid arteries. Of all patients, 217 (34.2%) were asymptomatic (434 carotid arteries); 417 (65.7%) had a history of an ischemic event (834 carotid arteries). There were 211 patients that had symptomatic carotid artery plaques, using the aforementioned criteria (50.6% of all symptomatic patients, 33.2% of all patients); 211 such plaques were analyzed, as they were unilateral. One-hundred and thirteen patients (17.8%) had imaging evidence of carotid IPH compared to 521 patients without (82.2%). The mean age of patients with carotid IPH was 71.4 years (SD = 9.9), compared to a mean age of 62.8 years (SD = 15.8) for patients without carotid IPH (*P* ≤ 0.0001).

### Multiple Logistic Regression Analysis

These data are summarized in Table 1. All 634 patients were included in this analysis. Variables that were independently-associated with carotid IPH included increasing age (OR: 1.04; 95% CI: 1.02–1.06; *P* = 0.001), male sex (OR: 2.80; 95% CI:1.66–4.70; *P* ≤ 0.0001), increasing degrees of stenosis (OR: 1.02; 95% CI: 1.01–1.03; *P* ≤ 0.0001) and hyperlipidemia (OR: 2.44; 95% CI: 1.19–5.04; *P* = 0.02). There were no other independent associations with carotid IPH.

**Table 1:** Multiple regression analysis for independent associations with carotid intraplaque hemorrhage.

**Variable**	**Odds ratio [95% CI]**	***P*-value**
Age	1.04 [1.02–1.06]	0.001
Male sex	2.80 [1.66–4.70]	<0.0001
Degree of stenosis	1.02 [1.01–1.03]	<0.0001
Coronary artery disease	0.96 [0.58–1.60]	0.88
Hypertension	0.97 [0.55–1.71]	0.91
Hyperlipidemia	2.44 [1.19–5.04]	0.02
Diabetes Mellitus	1.12 [0.66–191]	0.67
Obstructive sleep apnea	0.90 [0.52–1.54]	0.70
Ever smoker	1.06 [0.66–1.72]	0.79

*CI, confidence interval*.

### Stenosis and IPH Across Age Categories

Data for all, asymptomatic and symptomatic patients are summarized in Tables 2–4, respectively. In all patients, the severity of stenosis was found to increase with advancing age (*P* ≤ 0.0001). An increase in the prevalence of IPH was observed with increasing age (*P* = 0.0002), and each age group above 50 years had a statistically significant higher prevalence of IPH as compared to the 18–50 age group (*P* ≤ 0.05 for all comparisons). Similar trends were observed for asymptomatic patients in terms of increasing severity of stenosis and the prevalence of IPH with advancing age, though neither reached statistical significance (*P* = 0.07 and 0.14, respectively). However, all age groups above 60 years had a significantly higher prevalence of IPH when compared to the 18–50 age group (*P* ≤ 0.05). In patients with symptomatic carotid plaques, there was no significant difference in the severity of stenoses across age categories (*P* = 0.54), nor was there a significant difference in the prevalence of IPH (*P* = 0.32) (Table 3). When compared to the 18–50 age category, however, each group above 50 years of age had a significantly higher prevalence of IPH (*P* ≤ 0.05).

**Table 2:** All patients.

	**Age group**					
**Degree of**	**18–50 years**	**50–59**	**60–69**	**70–79**	**80+**	***P*-value**
**stenosis in %**	**(*N* =216)**	**(*N* = 212)**	**(*N* = 300)**	**(*N* = 344)**	**(*N* = 196)**	
<30	189 (87.5)	162 (76.4)	213 (71.0)	233 (69.8)	130 (66.3)	<0.0001
30–49	6 (2.8)	13 (6.1)	19 (6.3)	17 (5.1)	13 (6.6)	
50–69	10 (4.6)	13 (6.1)	35 (11.7)	45 (13.5)	20 (10.2)	
>70	11 (5.1)	24 (11.3)	33 (11.0)	49 (14.7)	33 (16.8)	
Intraplaque hemorrhage	2 (0.9)	16 (7.5)^*^	30 (10.0)^*^	42 (12.6)^*^	33 (16.8)^*^	0.0002

*N = number of carotid arteries*.

* *Statistically significant higher proportion compared to 18–50 age group*.

**Table 3:** Asymptomatic patients.

	**Age group, no. of arteries (%)**					
**Degree of**	**18–50 years**	**50–59**	**60–69**	**70–79**	**>80**	***P*-value**
**stenosis**	**(*N* = 146 total)**	**(*N* = 88 total)**	**(*N* = 88 total)**	**(*N* = 86 total)**	**(*N* = 26 total)**	
<30%	135 (92.5)	79 (89.8)	70 (79.5)	70 (81.4)	21(80.8)	0.07
30–49%	4 (2.7)	1 (1.1)	1 (1.1)	3 (3.5)	1 (3.8)	
50–69%	4 (2.7)	3 (3.4)	6 (6.8)	7 (8.1)	0 (0.0)	
>70%	3 (2.1)	5 (5.7)	11 (12.5)	6 (7.4)	4 (15.4)	
Intraplaque hemorrhage	1 (0.7)	2 (2.3)^†^	5 (5.7)^*^	7 (8.1)^*^	3 (11.5)^*^	0.14

*N = number of carotid arteries*.

* *Statistically significant higher proportion compared to 18–50 age group*.

† *No difference between 18 and 50 age group, or any other age group*.

**Table 4:** Patients with symptomatic carotid plaques.

	**Age group, no. of patients (%)**					
**Degree of**	**18–50 years**	**50–59**	**60–69**	**70–79**	**>80**	***P*-value**
**stenosis**	**(*N* = 20 total patients)**	**(*N* = 43 total patients)**	**(*N* = 49 total patients)**	**(*N* = 59 total patients)**	**(*N* = 40 total patients)**	
<30%	14 (70.0)	24 (55.8)	23 (49.9)	27 (45.8)	20 (50.0)	0.54
30–49%	1 (5.0)	5 (11.6)	3 (6.1)	3 (5.1)	4 (10.0)	
50–69%	2 (10.0)	7 (16.3)	14 (28.6)	11 (18.6)	7 (17.5)	
>70%	3 (15.0)	7 (16.3)	9 (18.4)	18 (30.5)	9 (22.5)	
Intraplaque hemorrhage	0 (0.0)	9 (20.9)^*^	17 (34.7)^*^	18 (30.5)^*^	13 (32.5)^*^	0.32

* *Statistically significant higher proportion compared to 18–50 age group, but no difference between groups*.

## Discussion

The results from this study indicate that MRA evidence of carotid IPH is independently-associated with increasing age. Furthermore, IPH seems to be rare among patients under 50 years of age regardless of symptomatic status. In this same respect, 50 years of age seems to be an approximate threshold at which IPH becomes more prevalent. These findings carry important implications in terms of utilizing MR carotid plaque imaging to assess for IPH in various age populations.

Among all patients, IPH was rarely noted in patients younger than 50 years of age. Symptomatic carotid plaques, in particular, never had IPH in adult patients under 50. Together, these findings suggest that younger patients may be less likely to benefit from plaque imaging than those over 50 years old. These results are corroborated by Singh et al. (12) who found that only 2 out of 63 patients (3.2%) with IPH were between the ages of 45–54, while 11, 14. and 36 patients were 55–64, 65–74, and >75 years, respectively. Moreover, a multitude of studies involving both symptomatic and asymptomatic patients with IPH have reported mean ages well-above 50 years of age further suggesting that IPH has an age-related dominance in patients above the age of 50 regardless of symptomatic manifestation (4, 5, 13, 14).

This study's results also fit with what is known about carotid artery plaque in general. The development of atherosclerotic plaques is a progressive process that occurs over decades resulting in plaques with higher histological grades and worsening severities of luminal stenosis (15). Plaques in younger patients are likely lower-grade lesions that are presumably less susceptible to hemorrhage. Furthermore, substantial carotid artery plaque is infrequent in patients under 50, and seems to increase in prevalence around this age (16). As such, a similar age threshold in IPH is therefore plausible. With advancing age, progressive plaques are likely to become increasingly stenotic in addition to assuming higher pathological grades, therefore predisposing to IPH. It is therefore unsurprising that both age and the degree of stenosis were found to be associated with the presence of IPH.

Neurologic events among younger patients are commonly due to embolic strokes of undetermined source (ESUS) (17–19). Recently, carotid artery atherosclerotic disease has been recognized as a potential embolic source in ESUS patients (20). Intriguingly, the presence of IPH in less stenotic carotid arteries has been associated with ipsilateral ischemic events in ESUS patients, suggesting that IPH specifically may be an underlying cause of ESUS (21). Our results, however, unfortunately suggest that IPH will be found infrequently on dedicated carotid plaque sequences in patients under 50 years old with ESUS.

Our data should be interpreted in context of the limitations of the study. The retrospective nature imposes the risk of selection bias unto our results. Patients with hemorrhagic carotid artery plaque may be asymptomatic and therefore may not have had any prior imaging studies performed. Therefore, these patients may be under-represented in our population. We used a broad categorization method for grouping patients with ischemic events, and the potential association of IPH with each individual disease entity (stroke, TIA, RAO, amaurosis) among certain age categories warrants further investigation. Multiple radiologists provided reports on plaque imaging studies that were included which may have resulted in heterogeneity among imaging study interpretations. We utilized a definition of IPH as being an intralesional signal intensity of 150% of the ipsilateral sternocleidomastoid muscle in accordance with prior reports (4, 5). Other reports, however, have utilized 200% as a cutoff intensity for the definition of IPH, which may improve specificity (22).

In conclusion, carotid atherosclerotic disease is a progressive process that occurs over decades and may be marked by IPH. Detection of IPH on carotid plaque imaging steadily increases after age 50 and is more frequent in symptomatic patients. Therefore, carotid plaque imaging has high diagnostic yield in the work up of cerebrovascular events in patients over 50 years of age. In patients younger than 50, IPH is rarely detected with MRA plaque imaging and more research is warranted to identify new imaging targets of plaque vulnerability.

## Data Availability Statement

The raw data supporting the conclusions of this article will be made available by the authors, without undue reservation.

## Ethics Statement

The studies involving human participants were reviewed and approved by Mayo Clinic Institutional Review Board. The patients/participants provided their written informed consent to participate in this study.

## Author Contributions

AL: data gathering, data analysis, manuscript writing, editing, and submission. JB, WB, and LS: conception, data analysis, manuscript editing. GL: data analysis, manuscript editing. JH: conception, data gathering, data analysis, and manuscript editing. DN: conception, data analysis, manuscript editing, and study supervision. All authors contributed to the article and approved the submitted version.

## Conflict of Interest

The authors declare that the research was conducted in the absence of any commercial or financial relationships that could be construed as a potential conflict of interest.

## Abbreviations

ESUS: embolic strokes of undetermined source
IPH: intraplaque hemorrhage
MPRAGE: Magnetization Prepared-Rapid Gradient Echo
NASCET: North American Symptomatic Carotid Endarterectomy Trial
RAO: retinal artery occlusion
TIA: transient ischemic attack.
